# Circulating tumor DNA (ctDNA) serial analysis during progression on PD-1 blockade and later CTLA-4 rescue in patients with mismatch repair deficient metastatic colorectal cancer

**DOI:** 10.1136/jitc-2021-003312

**Published:** 2022-01-31

**Authors:** Pashtoon Murtaza Kasi, Griffin Budde, Michael Krainock, Vasily N Aushev, Allyson Koyen Malashevich, Meenakshi Malhotra, Perry Olshan, Paul R Billings, Alexey Aleshin

**Affiliations:** 1 Department of Oncology/Hematology, Division of Internal Medicine, Weill Cornell Medicine/New York Presbyterian Hospital, New York, New York, USA; 2 Natera Inc, San Carlos, California, USA; 3 Scientific Affairs, Natera Inc, San Carlos, California, USA

**Keywords:** biomarkers, tumor, immunotherapy, tumor biomarkers, combined modality therapy

## Abstract

Immune checkpoint inhibitors have shown great promise in treating patients with mismatch repair deficient/microsatellite instability high (dMMR/MSI-H) colorectal cancer (CRC). Although single-agent pembrolizumab has been approved for first-line treatment of dMMR/MSI-H metastatic CRC, combination therapy with cytotoxic T-lymphocyte-associated protein-4 (CTLA-4) inhibition (ipilimumab/nivolumab) has reported higher response rates. It is unclear whether patients who progress on PD-1 inhibition will respond to CTLA-4 blockade. Here, we report a case series of three patients with dMMR/MSI-H mCRC, where a durable and ongoing response to nivolumab with ipilimumab was achieved after initial progression with pembrolizumab monotherapy. Blood-based biomarkers such as carcinoembryonic antigen and CA 19-9 were employed to assess treatment response and monitor disease progression along with circulating tumor DNA (ctDNA). Our findings indicate ctDNA’s potential to accurately monitor response to therapy and detect disease progression, as validated by standard imaging. This case series demonstrates that CTLA-4 rescue is worthy of additional investigation as a treatment strategy after progression on PD-1 blockade in patients with dMMR/MSI-high mCRC. Our data support the utilization and expansion of clinical studies with combination therapies and using ctDNA kinetics as early dynamic marker for therapy response assessment.

## Background

Immune checkpoint inhibitors (ICIs) have shown clinical benefit in patients with metastatic mismatch repair deficient/microsatellite instability-high (dMMR/MSI-H) colorectal cancer (CRC).^1^ In 2017, pembrolizumab became the first ICI to receive United States Food and Drug Administration (FDA) approval for dMMR/MSI-high solid tumors, and later, nivolumab was approved for CRC patients who had progressed on fluoropyrimidine, oxaliplatin, and irinotecan. In 2018, ipilimumab was approved for use in combination with nivolumab. Most recently (June 2020), pembrolizumab received FDA approval as first-line treatment for patients with unresectable or metastatic dMMR/MSI-H mCRC after the landmark KEYNOTE-177 trial results.^2 3^ These studies have laid the foundation for a deeper exploration of ICI use in this population. However, a proportion of dMMR/MSI-H CRC tumors did not attain long-term clinical benefit from ICI treatment. In the KEYNOTE-177 trial, one-third of patients with dMMR/MSI-H mCRC tumors (29.4%) had progressive disease as the best response with pembrolizumab.^2^

The addition of the cytotoxic T-lymphocyte-associated protein-4 (CTLA-4) inhibitor, ipilimumab to anti-PD-1/L1 monotherapy is currently being evaluated for use in cancers which have failed programmed cell death protein/ligand-1 (PD-1/L1) inhibition alone.^4^ While combination therapy with CTLA-4 and PD-1/L1 is listed as a treatment option in the NCCN CRC guidelines (V.3.2021),^5^ ipilimumab has not been comprehensively studied as a second line of therapy in tumors that do not respond to PD-1/L1 inhibition. It is also unclear if patients with dMMR/MSI-H mCRC will respond to ‘CTLA-4 rescue’ after progression on anti-PD-1/PD-L1 drugs. Thus far, only a few studies have reported the value of ‘immunotherapy after immunotherapy’ in patients with dMMR/MSI-H tumors.^6 7^ Here, we present a case series of three patients with dMMR/MSI-H mCRC, where a favorable, durable and ongoing response to nivolumab with ipilimumab was achieved after initial progression on pembrolizumab monotherapy.

## Case series

Three dMMR/MSI-H mCRC patients were enrolled into an expanded access program for analysis of their circulating tumor DNA (ctDNA), using a personalized, tumor-informed assay (Signatera bespoke mPCR-NGS assay). The assay is validated to detect ctDNA with high sensitivity down to 0.01% tumor fraction. The assay tracks 16 tumor-specific, clonal, somatic, single nucleotide variants (SNVs) in plasma, based on up front whole exome sequencing of the tumor tissue and matched normal blood.^8^ Plasma samples with ≥2 SNVs detected above a predefined confidence threshold were considered ctDNA-positive and ctDNA levels were reported in mean tumor molecules per mL (MTM/mL) of plasma. The MMR, MSI and tumor mutational burden status of the patients and specific point mutations found using the FoundationONE panel are represented in table 1 and online supplemental figure 1. We present serial ctDNA results from these patients, taken while they were receiving ICI therapy. All three patients progressed on pembrolizumab and then were given nivolumab with ipilimumab (‘CTLA-4 rescue’). Tumor response to the treatment regimen was monitored by radiological imaging. Longitudinal measurements of carcinoembryonic antigen (CEA) and carbohydrate antigen (CA) 19-9 levels were also collected to assess treatment response and monitor for disease progression.

10.1136/jitc-2021-003312.supp1 Supplementary data

**Table 1:** NGS results of patients

	Case 1A*	Case 1B*	Case 2	Case 3
Mismatch repair (MMR) status				
MMR status	Deficient	Deficient	Deficient	Deficient
Microsatellite instability (MSI) status				
CGP-derived MSI (FoundationONE)	MSI-H	MSI-H	Could not be determined	Could not be determined
WES-derived MSI (MANTIS algorithm ran after Natera WES)	MSI-H	Sample not available	MSI-H	MSI-H
Tumor mutational burden (TMB), Muts/Mb				
TMB, CGP-derived (FoundationONE)	33	64	281	20
TMB, WES-derived (Natera WES)	43	Not available	351	31
Point mutations (FoundationONE panel)				
*BRAF*	Wild-type	Wild-type	Wild-type	*V600E*
*KRAS*	Wild-type	Wild-type	*A59T*	Wild-type
*NRAS*	Wild-type	Wild-type	Wild-type	Wild-type
*MMR-*related genes	MSH6 R248fs*8	*MSH6* R248fs*8	*MLH1 R265C,* MSH3 E512*, MSH6 E368*, E1234*	Wild-type
*BRCA/DNA-*repair-related genes	FANCA P1324fs*39	FANCA P1324fs*39, ATM E1892*, ATM F61fs*15	ATME 522*, *BRCA1 R1699W*	Wild-type
*ARID1A*	*Wild-type*	S254fs*146	R693*	Wild-type

*The first patient as noted had NGS testing done twice. Initially (case 1A) was on a colonoscopy biopsy of the primary tumor before chemotherapy and immunotherapy exposure. The second sample was a supraclavicular lymph node biopsy post-PD-1 progression. While there could be inherent differences both intratumoral (primary vs metastatic) and temporal (over time or secondary to treatment), these cannot be attributed to treatment alone.

CGP, comprehensive genomic profiling; MSI-H, MSI high; WES, whole exome sequencing.

## Case #1

**Figure 1:**
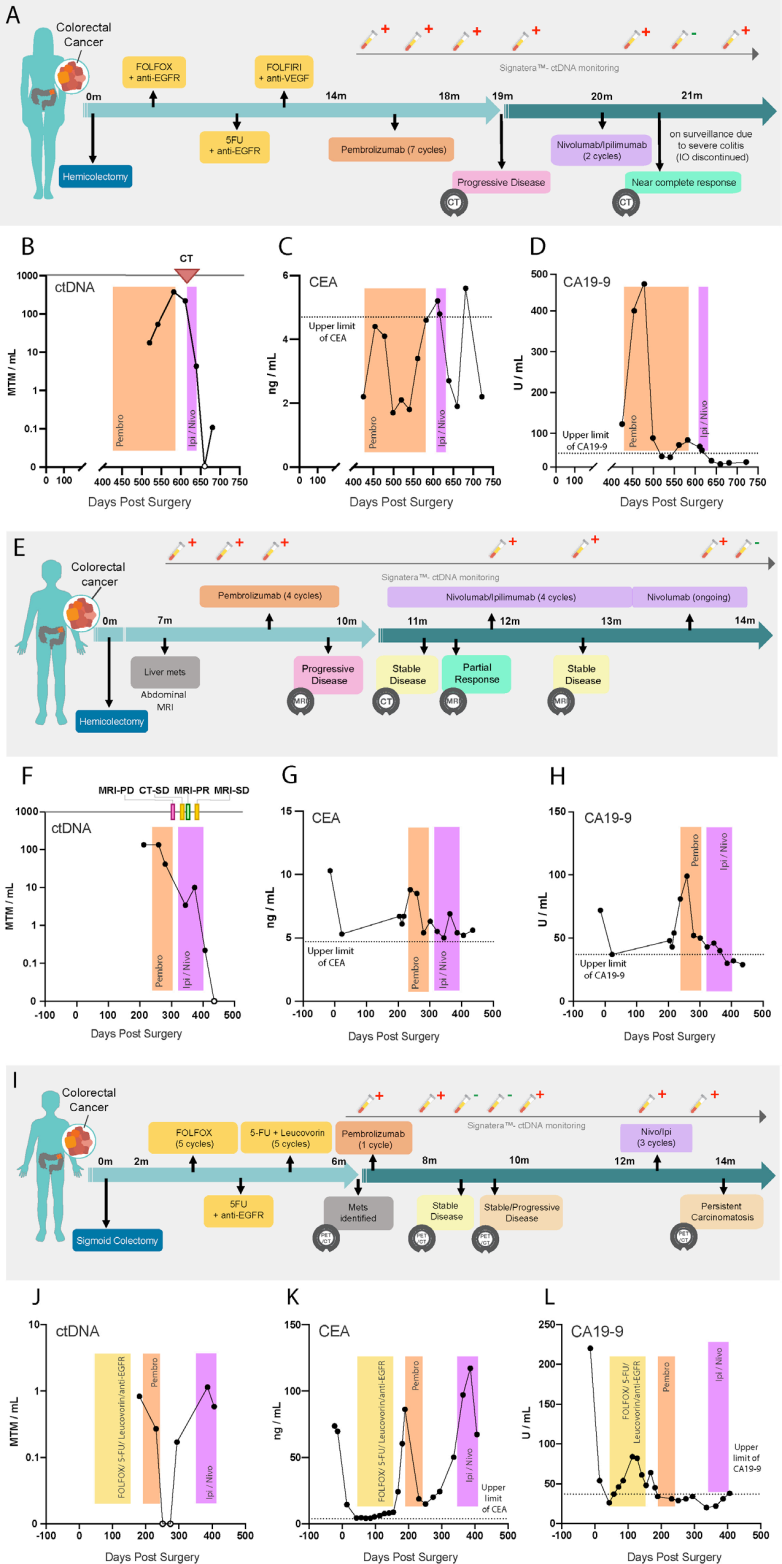
Clinical course and ctDNA, CEA and CA 19-9 monitoring. (A–D) Case #1, (E–H) Case #2, and (I–L) Case #3 patient clinical course and biomarker levels over time are represented. (A, E, I) Patient plots, providing details on the timeline of treatments administered, PET/CT scanning, and ctDNA monitoring. (B, F, J) ctDNA, (C, G, K) CEA and (D, H, L) CA 19-9 levels over time are represented, measured in number of days since surgical resection of the primary tumor. PET and CT scans, relapse, surgery, and therapeutic treatment windows are represented. CA, carbohydrate antigen; CEA, carcinoembryonic antigen; ctDNA, circulating tumor DNA; MTM, mean tumor molecules; PD, progressive disease; PET/CT, positron emission tomography/computed tomography;; PR, partial response; SD, stable disease 5FU, 5-fluorouracil,

A 61-year-old woman, pT2N1M1, presenting with near-complete bowel obstruction, underwent an upfront right hemicolectomy (figure 1A). Over 15 months, she was treated with FOLFOX with anti-EGFR, followed by 5FU (5-Fluorouracil) with anti-EGFR, then FOLFIRI with anti-VEGF. On progression, she underwent seven cycles of pembrolizumab (all patients included in this study received a standard dose (200 mg every 3 weeks) unless otherwise specified) for ~4 months. During this time, blood was collected and monitored for ctDNA, CEA, and CA 19-9. During pembrolizumab treatment, she tested positive for ctDNA three times, with levels increasing from 17.64 to 51.03 to 375.67 MTM/mL, respectively (figure 1B). In contrast, CEA remained below the upper limit of normal throughout pembrolizumab therapy, while CA 19-9 levels fluctuated between normal and elevated values (figure 1C, D). CT scan performed after pembrolizumab confirmed disease progression, demonstrating widespread lymph node metastasis with left subclavian, axillary, and retrocaval adenopathy. Ultrasound guided biopsy of a left cervical lymph node was performed, with pathology demonstrating metastatic adenocarcinoma. Given the patient’s progression on PD-1 blockade, combination treatment of nivolumab with low dose ipilimumab (1 mg/kg administered once every 3 weeks; also referred to as IPI-1 or IPI-light) was initiated and continued for two cycles. Following this, ctDNA levels sharply declined from 217.03 to 4.37 MTM/mL, eventually becoming undetectable. A repeat CT scan revealed a near complete response to CTLA-4 rescue, corroborating the undetectable ctDNA status. Unfortunately, the patient developed severe colitis, so dual checkpoint inhibition was stopped after two cycles. Following cessation of immunotherapy, ctDNA was again detected in the blood at 0.12 MTM/mL. CEA initially increased during this time, but returned to normal, and CA 19-9 remained in the normal range. However, radiological scans show no evidence of disease. As a result, the patient is on surveillance imaging with no therapy for 8 months.

## Case #2

A 69-year-old man underwent right hemicolectomy for pT3N0M1 stage IV CRC. (figure 1E). Seven months later, an abdominal MRI revealed metastatic disease in a cirrhotic liver, which was confirmed as adenocarcinoma by biopsy. The patient was then placed on 4 cycles of pembrolizumab. During this time, ctDNA, CEA, and CA 19-9 levels were elevated (134.29 MTM/mL; 8.5 ng/mL; and 99 U/mL, respectively) (figure 1F–H). MRI completed at the final cycle of pembrolizumab revealed disease progression. The patient was then placed on four cycles of nivolumab with IPI-light. CT scan performed after the first cycle of combination IO revealed stable disease, and a subsequent MRI showed a partial response. Similarly, although decreasing, ctDNA, CEA and CA 19-9 levels remained somewhat elevated (10.02 MTM/mL; 6.9 ng/mL; and 40 U/mL, respectively). A second MRI after the third cycle of combination IO revealed stable disease. Following this, the patient was maintained on q4 weekly nivolumab, with no evidence of progression or new sites of disease identified. A significant drop in ctDNA levels to undetectable was also observed during this period. At present, the patient continues to be on monthly nivolumab standard fixed dosing, planned for a total of 2 years.

## Case #3

A 60-year-old man underwent sigmoid colectomy for pT4aN2bM0 CRC (figure 1I). Two months postsurgery, the patient was initiated on adjuvant therapy. He underwent six cycles of FOLFOX, followed by five cycles of 5-FU with leucovorin. On therapy, ctDNA became detectable; therefore, a Positron Emission Tomography/Computed Tomography (PET/CT) scan was ordered, which identified peritoneal carcinomatosis with retroperitoneal and mesenteric lymph node metastases. The patient was subsequently initiated on pembrolizumab. He showed initial response, with a sharp decline in ctDNA and CEA levels (0.83 to 0.00 MTM/mL and 60.4 to 14.9 ng/mL, respectively), while CA 19-9 remained low (figure 1J–L). Two months after pembrolizumab, PET/CT scan showed stable disease, however, a repeat scan performed shortly after revealed progressive disease. Concurrently, ctDNA and CEA levels also increased (1.14 MTM/mL and 117.2 ng/mL, respectively). Owing to the biomarker fluctuations, as well as radiographic response and then later progression, the patient was administered with IPI-Light dosing alongside nivolumab. Although an initial response to combination IO was observed, the patient had to be initiated on systemic steroids, and combination IO was discontinued due to severe colitis. The ctDNA and CEA levels remained elevated (0.58 MTM/mL and 67.3 ng/mL, respectively). Two months later, PET/CT scan revealed persistent carcinomatosis (overall growth, but stable disease by RECIST). The patient continues to be monitored and has been placed back on single-agent IO with ongoing monthly nivolumab.

## Conclusions

Our case report is the first to test the efficacy of the CTLA-4 rescue strategy across multiple dMMR/MSI-H mCRC patients. At present, only two anecdotes exist as individual case reports that demonstrate the use of CTLA-4 rescue in MSI-high CRC.^6 7^ A phase II trial in melanoma reported 6 months of progression-free survival in 75% of patients after being treated with pembrolizumab with ipilimumab.^9^ Given that our cohort only includes three patients, future work exploring CTLA-4 rescue as a therapeutic strategy in larger dMMR mCRC cohorts is warranted.

Serum biomarkers for mCRC such as CA 19-9 and CEA are popular, however, these may not be ideal for monitoring poorly differentiated tumors, such as MSI-high CRC.^10^ MSI-high CRC reportedly produce significantly lower levels of serum CEA than well-differentiated tumors (33.90 ng/mL vs 387.66 ng/mL; p=0.03).^11^ Our recent study showed that MSI-high mCRC has distinctly low levels of both CEA and CA-19-9 compared with other mCRC subtypes, including MSS, which have up to eightfold greater levels of CA 19-9 and CEA.^12^ Though, our results indicate that both ctDNA and CEA tracked response to treatment, a greater degree of variability/fluctuation was observed with CEA levels when compared with changes in ctDNA. This signifies ctDNA is a reliable biomarker for monitoring disease status in patients with MSI-high CRC and can track response to immunotherapy at the molecular level. Furthermore, observed ctDNA trends predicted tumor responses weeks ahead of standard imaging, exhibiting ctDNA as a dynamic predictive marker.

Finally, even though nivolumab/ipilimumab is listed as a treatment option for dMMR/MSI-High tumors in addition to single agent pembrolizumab or nivolumab, it is not listed as an option post PD-1 progression. Our case series supports the inclusion of such combination therapies in clinical studies and using ctDNA as an early dynamic marker for assessment of therapeutic response.
