# Supplementary material for: Circulating tumor DNA (ctDNA) serial analysis during progression on PD-1 blockade and later CTLA-4 rescue in patients with mismatch repair deficient metastatic colorectal cancer
Source: J Immunother Cancer. 2022 Jan 31;10(1):e003312. doi: 10.1136/jitc-2021-003312 (PMC8804692; doi:10.1136/jitc-2021-003312)
Supplement: Supplementary data [file jitc-2021-003312supp001.pdf]

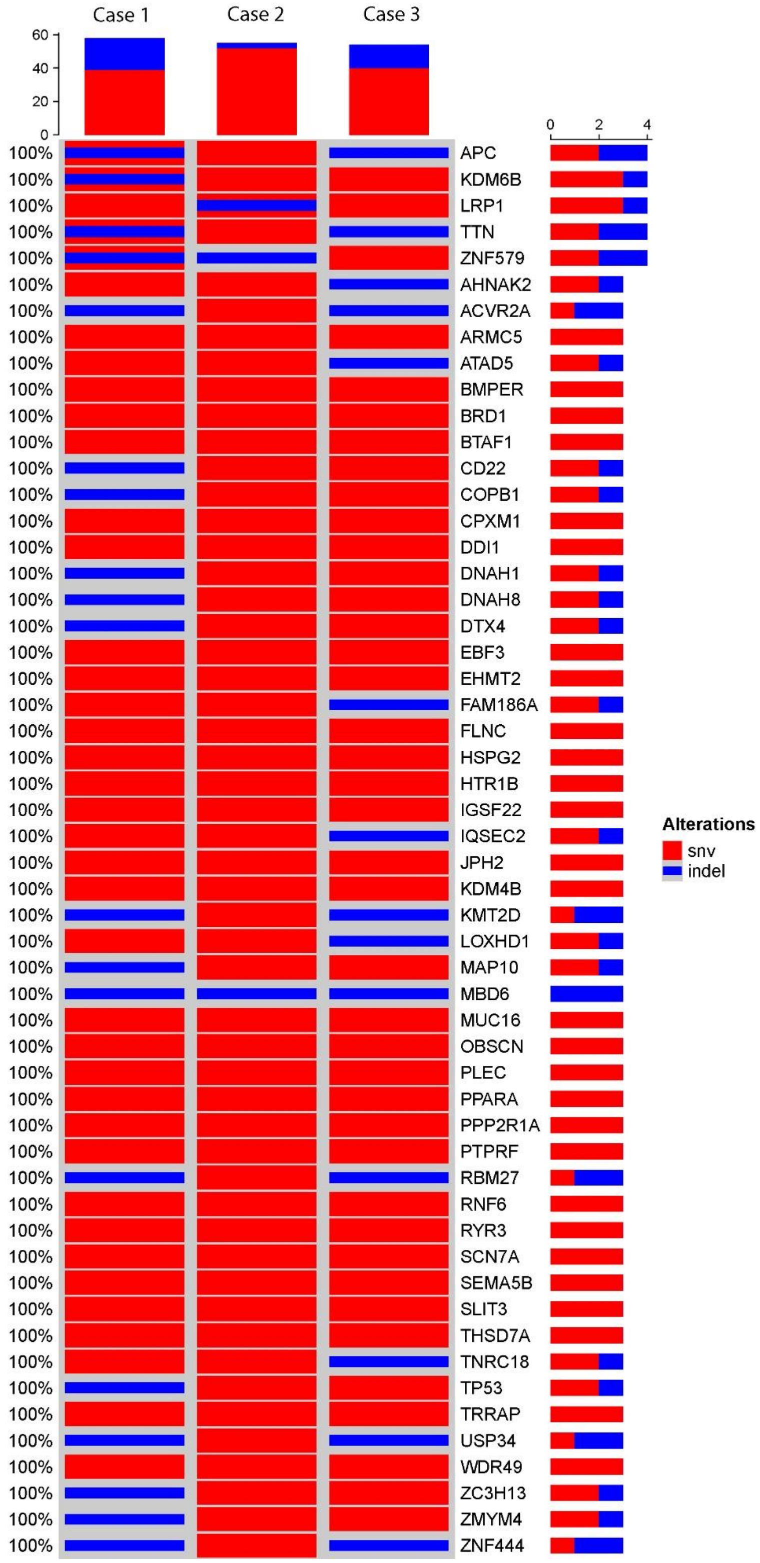

Case 1
Case 2
Case 3
60
40
20
0
0
2
4
100%
APC
KDM6B
LRP1
TTN
ZNF579
AHNAK2
ACVR2A
ARMC5
ATAD5
BMPER
BRD1
BTAF1
CD22
COPB1
CPXM1
DDI1
DNAH1
DNAH8
DTX4
EBF3
EHMT2
FAM186A
FLNC
HSPG2
HTR1B
IGSF22
IQSEC2
JPH2
KDM4B
KMT2D
LOXHD1
MAP10
MBD6
MUC16
OBSCN
PLEC
PPARA
PPP2R1A
PTPRF
RBM27
RNF6
RYR3
SCN7A
SEMA5B
SLIT3
THSD7A
TNRC18
TP53
TRRAP
USP34
WDR49
ZC3H13
ZMYM4
ZNF444
Alterations
snv
indel

Supplementary Figure 1: Oncoprint plot for 54 non-synonymous changes common for all three cases, each case shown by one lane. The upper bar plot shows the total number of alterations detected in each case. The right panel shows relative prevalence of alterations in each gene (for example, *APC* had both indel and SNVs for case #1, SNVs for case #2, indel for case #3 – thus total of 4 alterations is shown at the right barplot). All three cases (as noted in Table 1) were MMR-deficient, confirmed by immunohistochemistry, and MSI-high according to whole-exome sequencing of tumor tissue. Importantly, all cases also had high tumor mutational burden: 43, 351, and 31 mutations/Mb for cases #1, #2, and #3, respectively. With a wide spectrum of mutations found, 54 non-synonymous mutations were common for all three cases including genes commonly mutated in CRC, such as *APC* and *TP53*. Cases #1 and #2 had missense mutations in both *POLE* and *POLD1* genes. Case #2 also bore mutations *KRAS A59T* and *BRAF* P192S, while case #3 had *BRAF* V600E mutation. The tumor mutational burden (table 1) not surprisingly is different across different platforms.
